# Update on the collaborative interventions for circulation and depression (COINCIDE) trial: changes to planned methodology of a cluster randomized controlled trial of collaborative care for depression in people with diabetes and/or coronary heart disease

**DOI:** 10.1186/1745-6215-14-136

**Published:** 2013-05-11

**Authors:** Peter A Coventry, Karina Lovell, Chris Dickens, Peter Bower, Carolyn Chew-Graham, Andrea Cherrington, Charlotte Garrett, Chris J Gibbons, Clare Baguley, Kate Roughley, Isabel Adeyemi, Chris Keyworth, Waquas Waheed, Mark Hann, Linda Davies, Farheen Jeeva, Chris Roberts, Sarah Knowles, Linda Gask

**Affiliations:** 1 Greater Manchester Collaboration for Leadership in Applied Health Research and Care, Institute of Population Health and Manchester Academic Health Science Centre, University of Manchester, Manchester M13 9PL, UK; 2 School of Nursing, Midwifery & Social Work and Manchester Academic Health Science Centre, University of Manchester, Manchester M13 9PL, UK; 3 Peninsula College of Medicine and Dentistry, University of Exeter and Peninsula Collaboration for Leadership in Applied Health Research and Care (PenCLAHRC), Exeter, Devon EX1 2LU, UK; 4 IAPT North West Programme Field Lead, NHS North West, Manchester M60 7LP, UK; 5 Lancashire Care NHS Foundation Trust, Preston PR5 6AW, UK

**Keywords:** Depression, Diabetes, Coronary heart disease, Primary care, Collaborative care

## Abstract

**Background:**

The COINCIDE trial aims to evaluate the effectiveness and cost-effectiveness of a collaborative care intervention for depression in people with diabetes and/or coronary heart disease attending English general practices.

**Design:**

This update details changes to the cluster and patient recruitment strategy for the COINCIDE study. The original protocol was published in *Trials* (http://www.trialsjournal.com/content/pdf/1745-6215-13-139.pdf). Modifications were made to the recruitment targets in response to lower-than-expected patient recruitment at the first ten general practices recruited into the study. In order to boost patient numbers and retain statistical power, the number of general practices recruited was increased from 30 to 36. Follow-up period was shortened from 6 months to 4 months to ensure that patients recruited to the trial could be followed up by the end of the study.

**Results:**

Patient recruitment began on the 01/05/2012 and is planned to be completed by the 30/04/2013. Recruitment for general practices was completed on 31/10/2012, by which time the target of 36 practices had been recruited. The main trial results will be published in a peer-reviewed journal.

**Conclusion:**

The data from the trial will provide evidence on the effectiveness and cost-effectiveness of collaborative care for depression in people with diabetes and/or coronary heart disease.

**Trial registration:**

Trial registration number: ISRCTN80309252

## Update

### Introduction

The Collaborative Interventions for Circulation and Depression (COINCIDE) trial aims to investigate the effectiveness and cost-effectiveness of a collaborative care intervention for depression for people with diabetes and/or coronary heart disease [1]. The study is a cluster-randomised controlled trial conducted in English general practices. General practices were randomised to either a collaborative care arm or a usual care arm. The primary outcome is change in depression severity, as measured on the symptoms checklist-90 (SCL-90).

Eligible patients will be listed on Quality and Outcomes Framework disease registers for diabetes and/or coronary heart disease at the participating general practices. Due to poor detection rates and under-referral for treatment for depression in primary care in the UK, consenting patients will be screened for depression by the trial team using the Patient Health Questionnaire-9 (PHQ-9) on two occasions, two weeks apart. Patients will be considered to be depressed if they return PHQ-9 scores ≥10 on both occasions. Patients will be excluded from the study if they have a current diagnosis of dementia, are currently receiving a psychosocial intervention for depression, or treatment for drug or alcohol dependence.

### Study progress

#### Patient and GP practice recruitment

General practice recruitment began on 3 January 2012. The patient recruitment phase of the trial began when the first patient was recruited into the study on 1 May 2012. The average number of patients recruited per practice was assessed after the first eleven practices had finished recruitment. The average number of patients for these practices was 10 (range 4 to 22), below the target figure of 15 per practice.

The Trial Steering Committee (TSC) met on 17/10/12 when strategies for enhancing recruitment into the trial were discussed. It was agreed that the most efficient method to improve patient recruitment was to increase the number of general practices in the trial and to reduce the follow-up period. Ethical and research governance approval (see Amendment 9 from the NRES Committee North West - Preston, REF 11/NW/0742) was therefore sought to revise the sample size. To ensure that we retained power to detect a difference in the main outcome measure (SCL-90) we increased the number of clusters from 30 to 36. If we recruit 10 patients per practice then we anticipate a final sample size of 360. With an average of 10 patients over 36 practices (18 control/18 intervention) the study will have 79.1% power to detect a moderate standardised effect size of 0.4 at the 5% level of significance, allowing for an intraclass correlation (ICC) of 0.06.

GP and patient recruitment ran concurrently until 31 October 2012, when the target of 36 general practices was achieved.

### Follow-up

To take into account the additional time needed to recruit patients from six additional clusters, baseline patient recruitment was extended until 30 April 2013. Ethical and research governance approval (see Amendment 9 from the NRES Committee North West - Preston, REF 11/NW/0742) was granted to reduce the follow-up period from six to four months, owing to the need to complete all follow-up before the end-of-study date on 30 September 2013. Follow-up at four months post randomisation has been used in other depression trials in primary care and is the earliest time point that we might anticipate detecting a treatment effect [2].

## Discussion

Modifications to cluster and patient recruitment targets following feedback to the TSC have offered opportunities for this trial to retain adequate statistical power and deliver on all aims and objectives. This process underlines the importance of regular scrutiny of study progress by both the research and project management team and by independent monitors, such as a TSC [3].

## Abbreviations

COINCIDE: Collaborative Interventions for Circulation and Depression; ICC: intraclass correlation; PHQ: Patient Health Questionnaire; SCL: symptom checklist; TSC: Trial Steering Committee.

## Competing interests

PC, CG, CJG, CK, IA, KR, AC, FJ, CD, CCG declare they have no competing interests. PB is a paid scientific consultant to the British Association of Counselling and Psychotherapy.

## Authors’ contributions

PC is the Chief Investigator; designed the study and wrote the first draft of the protocol; revised and edited subsequent versions for publication. LG is the co-lead of the program and contributed to study design and co-authored the manuscript. CD contributed to study design and outcome selection and edited the manuscript. PB contributed to study design and co-authored the manuscript. CB contributed to the design of the training program and edited the manuscript. C CG contributed to the design of the process evaluation and edited the manuscript. CG contributed to the design of the training programme and edited the manuscript. CJG prepared the manuscript for publication and assisted with trial design and selection of outcome measures. KL co-authored the manuscript and contributed to the design of the training program. IA contributed to the design of the recruitment strategy and edited the manuscript. KR contributed to writing sections on therapeutic interventions and edited the manuscript. CK contributed to the writing the user engagement section and edited the manuscript. WW contributed to the design of the recruitment strategy for South Asian patients and translation of outcome measures. MH performed the sample size calculation and edited the manuscript. LD contributed to writing the health economic evaluation and edited the manuscript. FJ contributed to writing the health economic evaluation and edited the manuscript. CR contributed to the statistical analysis of the study. SK contributed to writing the process evaluation section. AC contributed to study design and is the Trial Manager. All authors edited the manuscript and read and approved the final manuscript.
